# Supplementary material for: Molecular Interaction Mechanism and Preservative Effect of Lactone Sophorolipid and Lactoferrin/β-Lactoglobulin Systems
Source: Foods. 2023 Apr 7;12(8):1561. doi: 10.3390/foods12081561 (PMC10137667; doi:10.3390/foods12081561)
Supplement: Supplementary file 1 [file foods-12-01561-s001.zip › foods-2280048-supplementary.pdf]

Supporting Information

For

Molecular Interaction Mechanism and Preservative Effect for the Systems of Lactone Sophorolipid and Lactoferrin/*β*-Lactoglobulin

Yanrong Chen, Mingyuan Li, Jing Kong, Jie Liu*, Qian Zhang*

School of Chemistry and Chemical Engineering, Liaocheng University, Liaocheng 252059, China

* Corresponding authors. liujielcu@126.com (J.L.) or zhangqianlcu@163.com (Z.Q.); Tel.: +86-635-8230614

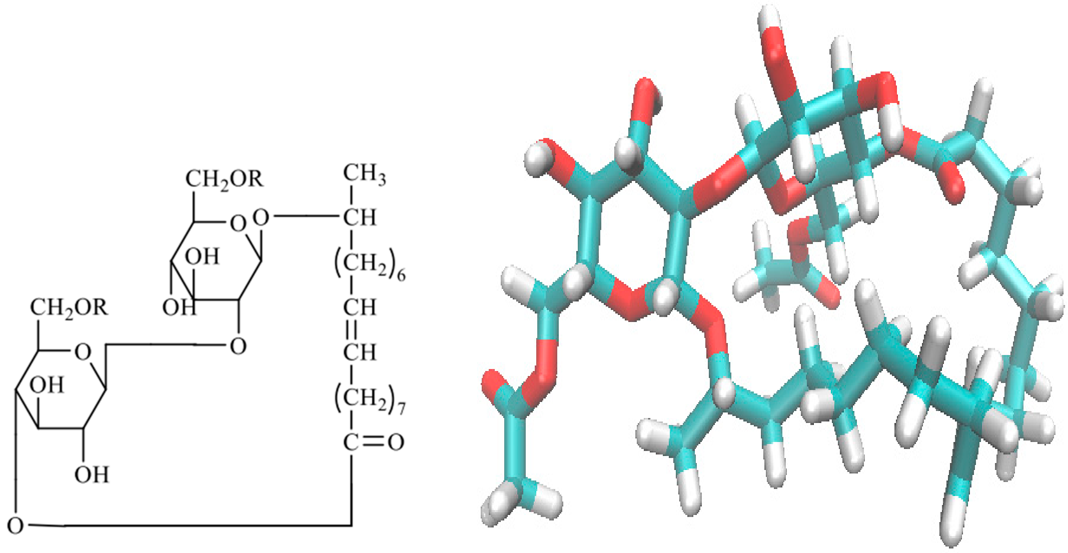

Figure S1 The structure of lactone sophorolipid (LSL).

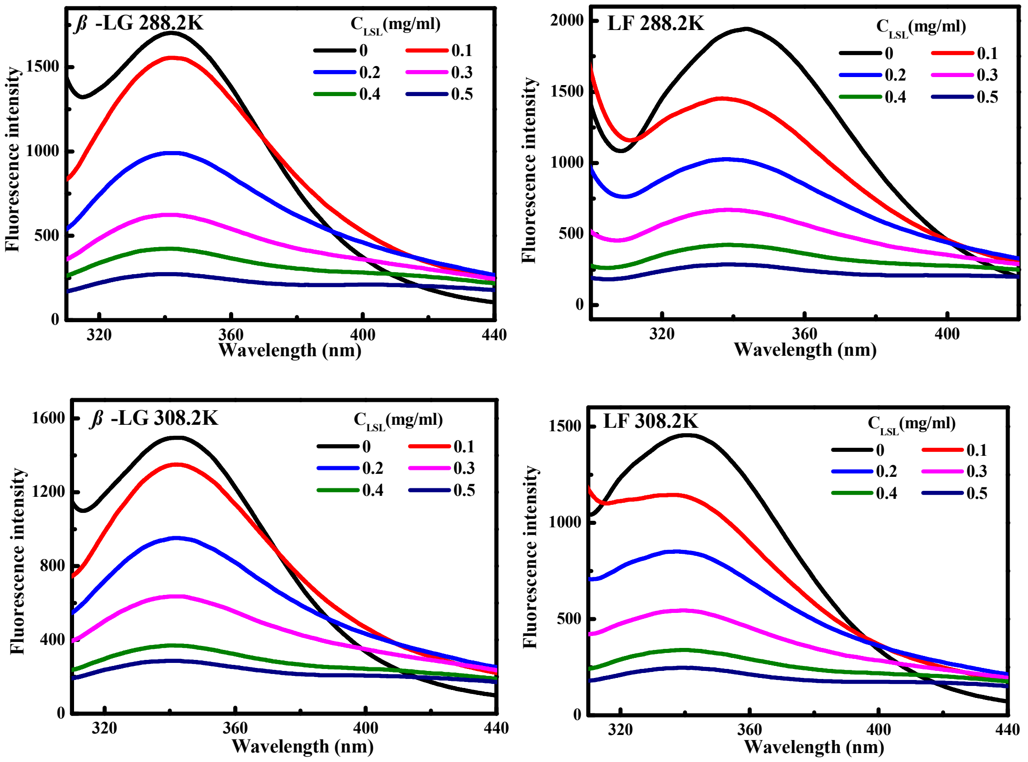

Figure S2 The fluorescence spectrum of β-LG/LF solutions with different LSL concentrations (0~0.5 g/L) at 288.2 K and 308.2 K.

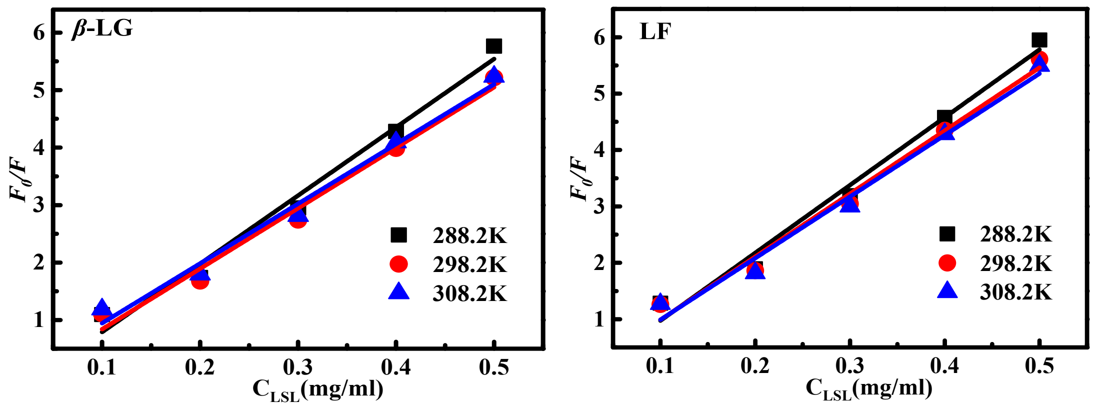

Figure S3 The Stern-Volmer plots of LSL quenching *β*–LG/LF at different temperatures.

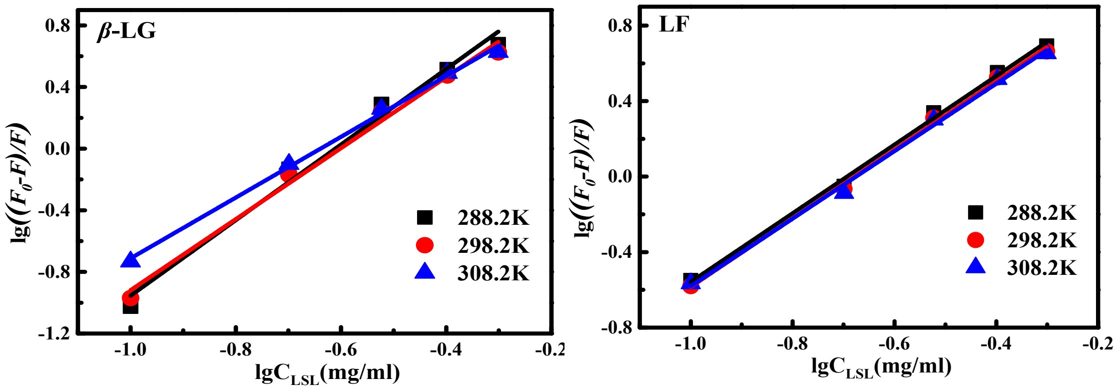

Figure S4 The double logarithmic plots of LSL quenching *β*–LG/LF at different temperatures.

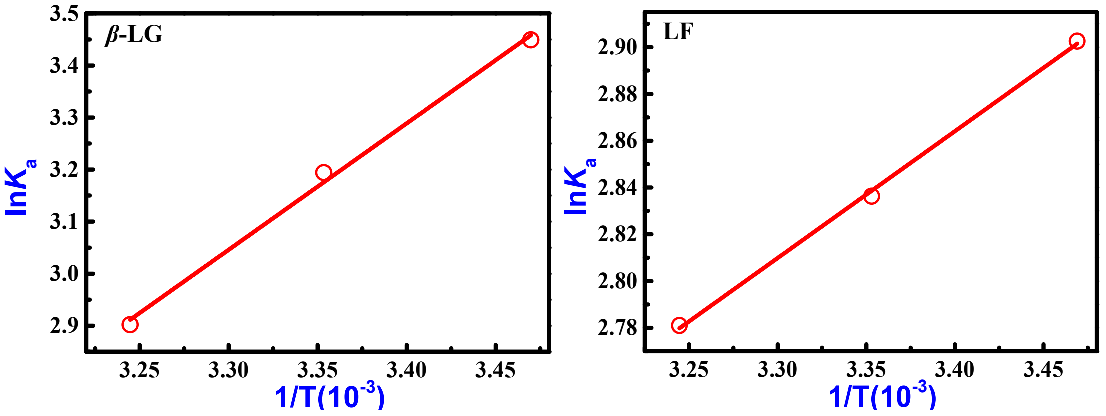

Figure S5 Van’t Hoff plots of the binding interaction for LSL with *β*–LG/LF.

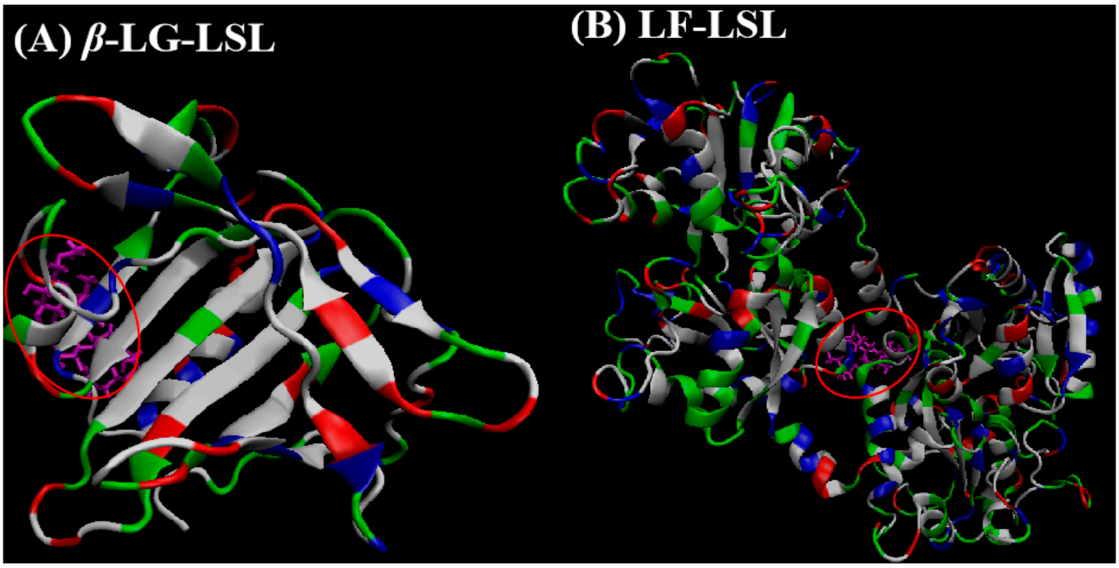

Figure S6 The molecular model of β-LG-LSL and LF-LSL.
